# A surgical simulator for peeling the inner limiting membrane during wet conditions

**DOI:** 10.1371/journal.pone.0196131

**Published:** 2018-05-14

**Authors:** Seiji Omata, Yusei Someya, Shyn’ya Adachi, Taisuke Masuda, Takeshi Hayakawa, Kanako Harada, Mamoru Mitsuishi, Kiyohito Totsuka, Fumiyuki Araki, Muneyuki Takao, Makoto Aihara, Fumihito Arai

**Affiliations:** 1 Department of Micro-Nano Mechanical Science and Engineering, Graduate School of Engineering, Nagoya University, Nagoya, Aichi, Japan; 2 Department of Precision Mechanics, Faculty of Science and Engineering, Chuo University, Bunkyo, Tokyo, Japan; 3 Department of Mechanical Engineering, School of Engineering, The University of Tokyo, Bunkyo, Tokyo, Japan; 4 Japan Science and Technology Agency (JST), Chiyoda, Tokyo, Japan; 5 Department of Ophthalmology, School of Medicine, The University of Tokyo, Bunkyo, Tokyo, Japan; University of Utah (Salt Lake City), UNITED STATES

## Abstract

The present study was performed to establish a novel ocular surgery simulator for training in peeling of the inner limited membrane (ILM). This simulator included a next-generation artificial ILM with mechanical properties similar to the natural ILM that could be peeled underwater in the same manner as in actual surgery. An artificial eye consisting of a fundus and eyeball parts was fabricated. The artificial eye was installed in the eye surgery simulator. The fundus part was mounted in the eyeball, which consisted of an artificial sclera, retina, and ILM. To measure the thickness of the fabricated ILM on the artificial retina, we calculated the distance of the step height as the thickness of the artificial ILM. Two experienced ophthalmologists then assessed the fabricated ILM by sensory evaluation. The minimum thickness of the artificial ILM was 1.9 ± 0.3 *μ*m (n = 3). We were able to perform the peeling task with the ILM in water. Based on the sensory evaluation, an ILM with a minimum thickness and 1000 degrees of polymerization was suitable for training. We installed the eye model on an ocular surgery simulator, which allowed for the performance of a sequence of operations similar to ILM peeling. In conclusion, we developed a novel ocular surgery simulator for ILM peeling. The artificial ILM was peeled underwater in the same manner as in an actual operation.

## Introduction

A macular hole is a foveal defect located at the center of the macula that can induce retinal tears and rhegmatogenous retinal detachments [1]. A harvested inner limiting lamina often includes an epiretinal membrane (ERM) on the inner limiting membrane (ILM) with adherent cells [2–5]. Treatment of the macular hole removes the ERM, and an ILM peeling procedure has been suggested to decrease the risk of retinal detachment [2]. In addition to removal of the ERM, peeling of the ILM improves the therapeutic effect for the macular hole [6–8].

An overload applied to the retina during ILM peeling can induce retinal detachment and macular edema. In this situation, an experienced surgeon is needed because novice surgeons lack the necessary technical skills and are inadequately prepared for this type of ocular surgery. In addition, an extracted eyeball cannot be used for training of ILM peeling because it is difficult to find natural ILMs and retinal detachment is likely to occur. Thus, a realistic artificial eye model is needed to simulate ILM peeling.

Although model-based surgical simulators such as the Kitaro WetLab (Frontier Vision Co., Ltd., Hyogo, Japan), Phake-i (Eye Care and Cure, Tucson, AZ, USA), and BIONIKO models (BIONIKO, Aventura, FL, USA) [9–11] have been developed, less is known regarding how to develop a realistic simulator for vitreoretinal surgery. Previously established technologies to simulate ILM peeling mainly utilize a quail eggshell membrane. This membrane is easy to obtain at low cost, but the reproducibility is low because of individual differences and because the film properties do not adequately mimic the natural ILM [12]. On the other hand, a virtual reality simulator such as the Eyesi (VRmagic, Mannheim, Germany) [13] has an ILM peeling task, and this simulator provides assessment and feedback of the task to the surgeon [13–15]. Although such devices are suitable for simulating surgery because of their high reproducibility and lack of ethical problems, obtaining three-dimensional data is often difficult, and extensive time is required to apply the new device to a computer. In the Phake-i, the ERM and ILM are equipped using polydimethylsiloxane (PDMS) thin films [10], although simulation of the ERM and ILM is poor. Moreover, because the Phake-i is not filled with liquid such as intraocular perfusion fluid, it does not exactly simulate actual surgery. Arai et al. fabricated an artificial ILM that could be peeled under dry conditions [16]. This system needed improvement, however, because it was significantly stiffer than a natural ILM and was characterized by high flexibility during peeling. Additionally, it could not be operated in water, and the peeled ILM easily reattached to the surrounding ILM, base substrate, and microforceps.

**Fig 1:**
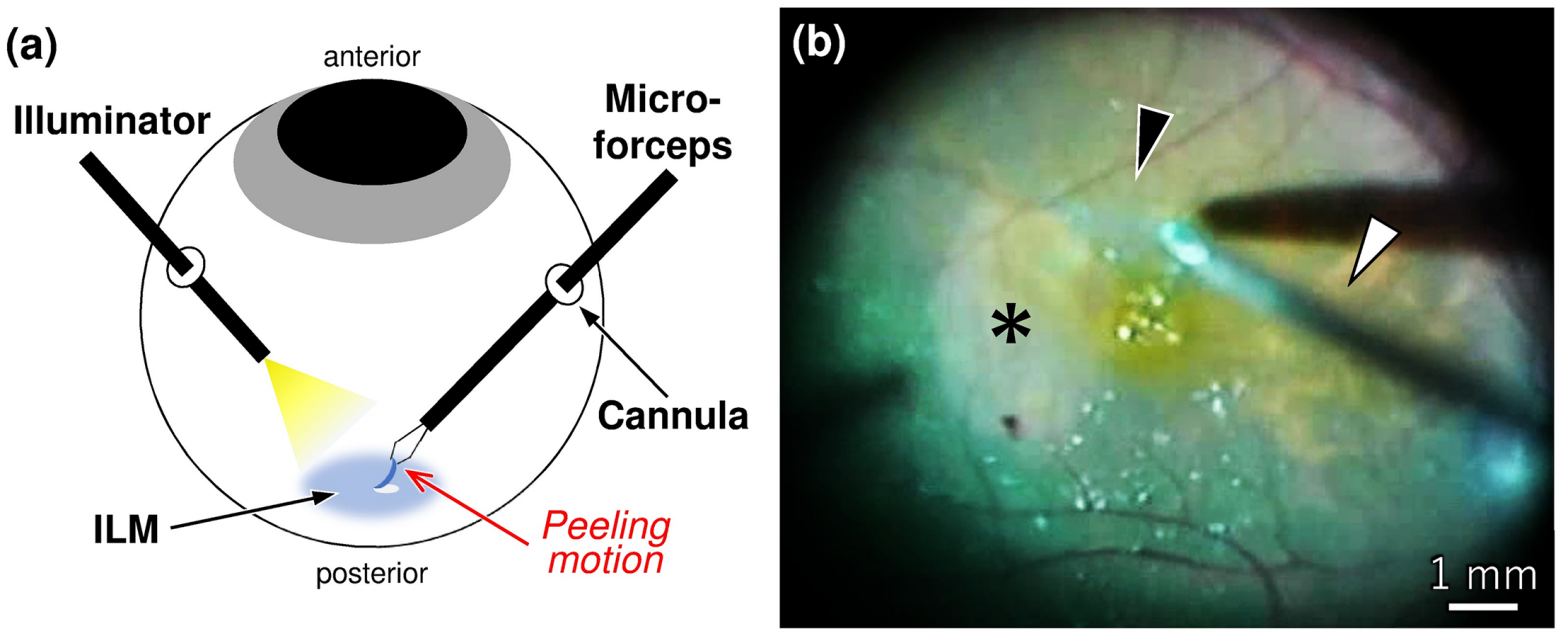
Peeling of the inner limiting membrane (ILM). (a) Schematic image of ILM peeling. (b) Bright image of peeling of the indocyanine green-stained natural ILM. Black arrowhead, peeled ILM; white arrowhead, microforceps; asterisks, retinal surface after removal of the ILM.

The purpose of the present study was to develop a next-generation artificial ILM with mechanical properties similar to those of the natural ILM that could be peeled underwater in the same manner as during an actual operation (Fig 1).

## Materials and methods

### Fabrication of eyeball

**Fig 2:**
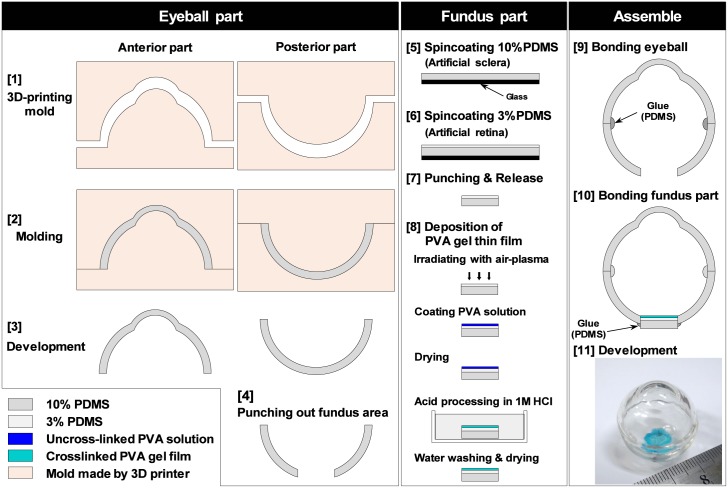
Fabrication process of a whole eyeball model equipped with an artificial ILM and retina.

Fig 2 shows the fabrication process of the artificial eye model used in this study. This model consisted of the fundus and eyeball parts. The fundus part was mounted in the eyeball part and consisted of an artificial sclera, retina, and ILM. The elasticity moduli of the sclera, retina, and ILM of the human eye are approximately 2000, 20, and 100 kPa, respectively [1, 17–20]. We used PDMS (Sylpot 184; Dow Corning Toray, Tokyo, Japan) to easily adjust the elastic modulus of the artificial model to simulate natural tissue. We mixed the base elastomer with the curing agent at either a 10:1 or 10:0.3 (g/g) ratio (10% PDMS and 3% PDMS) to model the sclera and retina, respectively. To fabricate the artificial ILM, a hydrogel obtained by chemically crosslinking poly(vinyl alcohol) (PVA) (saponification degree, 88%; Kuraray, Tokyo, Japan) was used as the main material and processed into a thin film [21–23].

We created the eyeball and assembled the ocular model containing the fundus and eyeball parts as shown in Fig 2:

1. Eye-shaped cast molds were made by a three-dimensional printer (Objet Eden250; Stratasys, Eden Prairie, MN, USA).
2. PDMS (10%) was poured into the cast mold and baked at 100°C for 2 hours.
3. The well-baked parts of the eyeball were removed for use as the artificial sclera.
4. A steel puncher was used to punch the bottom area of the posterior section to a diameter of 10 mm.
5. A 10% PDMS sheet was spin-coated and baked to a thickness of 1 mm to create an artificial sclera.
6. A PDMS sheet was spin-coated and baked to a thickness of 250 *μ*m to create an artificial retina.
7. The glass plate was removed by immersion in 70% Eta Cohol 7 (Sankyo Chemical Co., Ltd., Osaka, Japan) diluted with pure water.
8. The sheet was punched out to a diameter of 10 mm using a steel puncher.
9. Air plasma treatment was used to laminate an artificial ILM on the surface of the retina.
10. The anterior and posterior parts were bonded by baking at 100°C for 2 hours.
11. The fundus was bonded to the eyeball part and baked again.
12. The artificial eye was developed as a model for training in the ILM peeling procedure.

### Fabrication of fundus part

To fabricate the fundus, we placed a 10% PDMS solution on a glass plate coated with a lift-off resist (LOR-5B, MicroChem, Westborough, MA, USA) to more easily remove the glass plate, adjusted it to a 1-mm thickness, and then baked it in a heat chamber at 100°C for 2 hours to crosslink the PDMS as an artificial sclera. We spin-coated a 3% PDMS solution on the artificial sclera to a thickness of 250 *μ*m as an artificial retina and baked it again. We then punched out a sample sheet to a diameter of 10 mm using a steel puncher (FK-P17; Fukui Kiko Shokai, Osaka, Japan). To fabricate an artificial ILM with a chemically cross-linked PVA hydrogel, we prepared final concentrations of 100, 150, 200, and 250 mM PVA in water containing 500 mM glutaraldehyde (073-00536; Wako Pure Chemical Industries, Osaka, Japan), 1 mM gelatin (molecular weight, 2000; SCP-2000; Nitta Gelatin, Osaka, Japan), and 40 *μ*M Brilliant Blue FCF (027-12842; Wako Pure Chemical Industries). Four levels of polymerization (molecular weight) of PVA were used for sensory evaluation by an experienced investigator: 300 (13200), 100 (44000), 1700 (74800), and 2400 (105600). After irradiating the artificial retinal surface with air plasma treatment (Cute-1MR/R; Femto Science, Yongin, Republic of Korea) at 100 W for 5 minutes, we laminated 30 *μ*L of each PVA solution onto the plasma-treated surface. To gently dry the solution, we stored the sample in a refrigerator at 4–8°C overnight. It was then well-dried in a heat chamber at 70 or 120°C for 1 hour. After immersion in 1 M HCL solution at room temperature for 2 minutes and washing in pure water twice, we obtained the artificial ILM.

### Thickness measurement of artificial ILM

To measure the thickness of the fabricated ILM on the artificial retina, we moved the ILM to a glass plate [24], and the sample was swollen with pure water for 5 minutes. The ILM was then wiped to remove unnecessary moisture, and the thickness of the ILM was measured using a laser microscope (VK-9700; Keyence, Osaka, Japan).

### Sensory evaluation

Two experienced ophthalmologists assessed the fabricated artificial ILM by sensory evaluation. We then placed the fundus part on a plastic dish and added pure water to swell the artificial ILM. The resulting ILM was peeled using 25-gauge microforceps (Grieshaber Revolution 25-gauge asymmetrical forceps, 705.45P; Alcon Japan, Tokyo, Japan).

### Statistical analysis

The F-test for simple linear regression analysis with R language was used for curve fitting during assessment of the fabricated PVA film thickness. After fitting, we calculated the confidence coefficient *R*^2^ and then estimated Cohen’s *f*^2^ using *f*^2^ = *R*^2^/(1 − *R*^2^) [25]. A value of *f*^2^ > 0.35, *P*–*value* < 0.01, and power of (1 − *β*) > 0.8 were considered significant.

## Results

### Evaluation of thickness of artificial ILM

**Fig 3:**
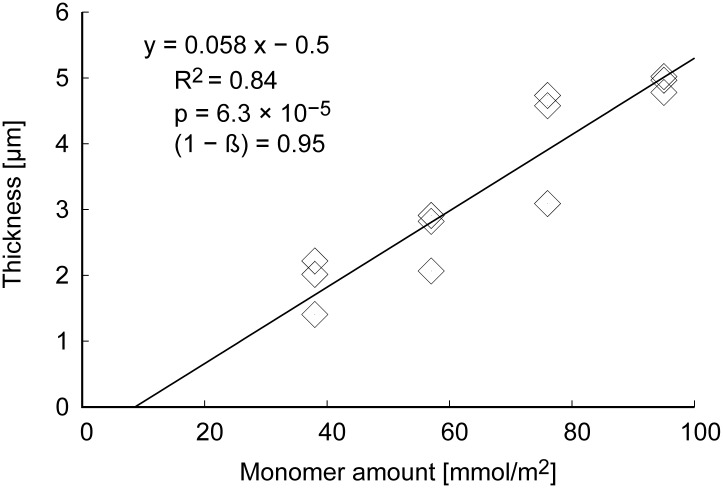
Thickness of artificial ILMs determined by a laser microscope in wet conditions.

Fig 3 shows the artificial ILM film thickness, demonstrating that the film thickness could be controlled by adjusting the amount of vinyl alcohol monomer per unit area. The thickness of a natural ILM increases to approximately 3 *μ*m with aging [1]. As a result, an estimated 43 mmol/m^2^ was needed to laminate a film thickness of approximately 3 *μ*m.

### Sensory evaluation

**Fig 4:**
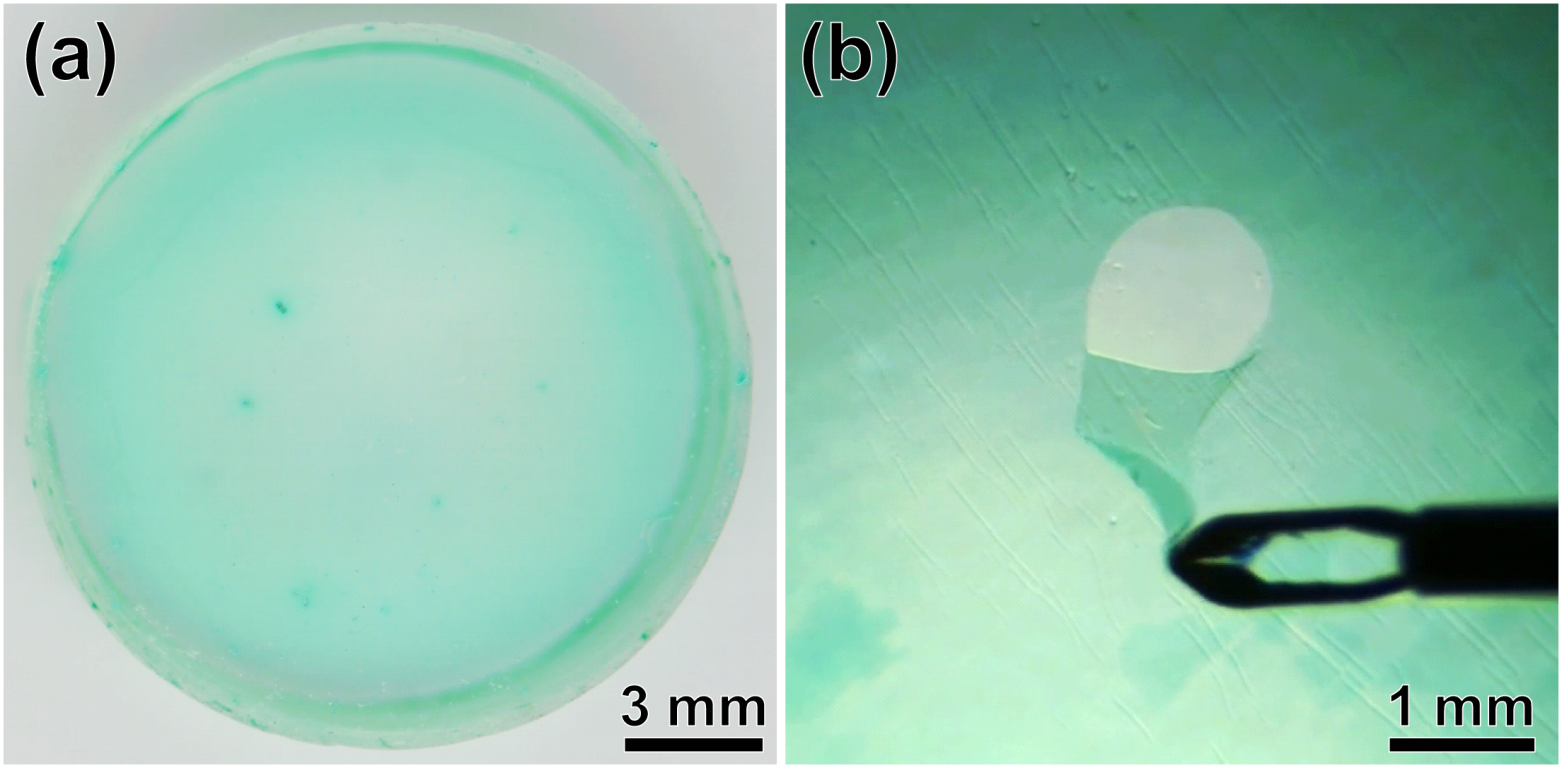
(a) Results of a fabricated artificial ILM and (b) a photograph of the peeling trial of the artificial ILM in water.

Fig 4a shows the results of fabricating the fundus, which exhibited success during the peeling task with the artificial ILM in pure water as shown in Fig 4b (S1 Movie). To evaluate whether this system is suitable for training of novice surgeons, Table 1 shows the sensory evaluation with different film thicknesses and molecular weights of the main material. The best evaluation was obtained with a thickness of 1.9 *μ*m and a degree of polymerization of 1000. When the main chain length was short using a degree of polymerization of 300, it was difficult to perform the peeling operation because the ILM was easily broken off. However, with degrees of polymerization of 1700 and 2000, the ILM was easier to stretch. The peeling action could be easily performed; the film was successfully peeled off without being torn, or the cut part was easily stretched even if it was torn.

**Table 1:** Result of sensory evaluation of artificial ILM in pure water.

Thickness (*μ*m)	Degree of polymerization			
	300	1000	1700	2400
1.9 ± 0.3	C A	A A	B A	B A
2.6 ± 0.4	–	A A	–	–
4.1 ± 0.4	–	C C	–	–
4.9 ± 0.1	–	C C	–	–

Grade for training of novice surgeons: A: suitable, B: slightly suitable, C: not suitable.

### Simulation of ILM peeling task with artificial eyeball

**Fig 5:**
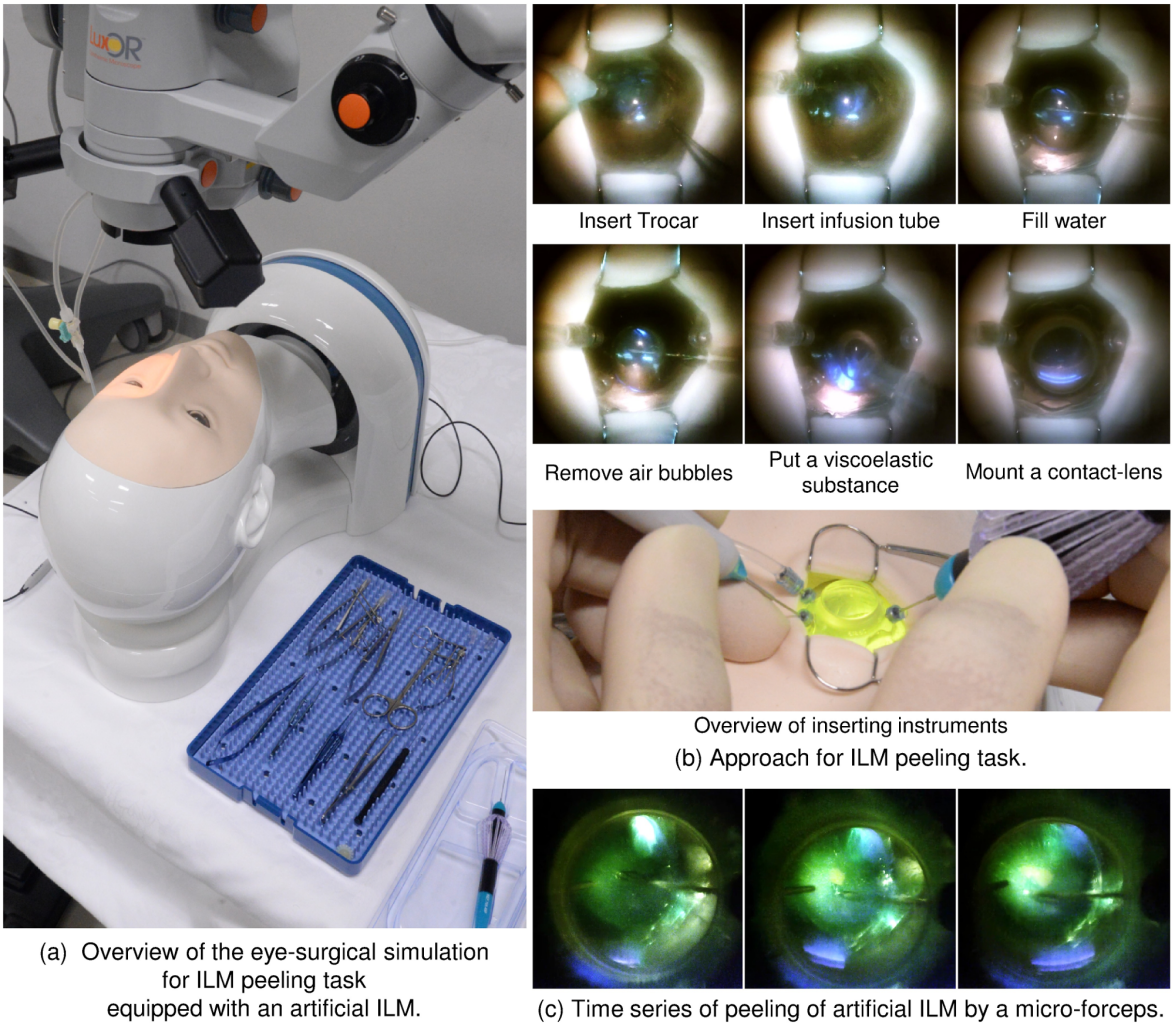
Photographs of the artificial ILM peeling task with an artificial eye mode.

Fig 5a shows simulated artificial ILM peeling with an artificial eye model mounted on an eye surgery simulator, which allowed for successful performance of the peeling task with the artificial ILM in water as shown in Fig 5b and 5c (S2 Movie). This eye model could be used with three trocars for inserting the infusion needle, illuminator, and microforceps and could be filled with water. When air bubbles entered, it was easy to remove them from the limbus with a needle. After completely filling the model with water, applying a viscoelastic substance, and inserting a contact lens, we could observe the fundus with an illuminator with either a white or yellow color. Finally, we were able to peel the artificial ILM with microforceps, showing that we succeeded in developing an ocular surgery simulator that facilitated performance of a sequence of operations similar to ILM peeling.

## Discussion

We have herein described the development of a novel eye model with an artificial ILM made of a hydrogel thin film in the fundus to simulate the ILM peeling procedure. Using PVA as the main polymer of the hydrogel, we succeeded in fabricating an artificial ILM. As a result, it was easy to adjust the degree of polymerization, the amount of PVA lamination, and the monomer density per unit area. We could then qualitatively adjust the film thickness. It was also possible to assess qualitative changes using sensory evaluations. The thickness of the natural ILM increases to a maximum of approximately 3 *μ*m with aging, and the thickness further increases in diabetic patients. Because we wanted to simulate the ILM peeling procedure as in actual patients undergoing the surgery, the minimum thickness of the artificial ILM was 1.9 *μ*m, making it possible to form a film suitable for training of novice surgeons. We showed that the ILM could be pinched even with microforceps. However, to show the bionic properties of the fabricated ILM, it was necessary to quantitatively evaluate the engineering parameters such as the elastic moduli of the harvested natural and artificial ILMs and the detachment force from the retina. The film thickness measurement results suggested that it was difficult to fix/chuck a thin film such as a natural or artificial ILM with a jig of a force-sensor system in the tensile and delamination tests. Thus, it was necessary to develop these evaluation systems so that the film could be precisely measured. In the present study, only a physical/design model for elastic modulus was considered, and it should be mentioned that ILM stripping involved many physical phenomena as indicated by the engineering parameters. Therefore, information about physical modeling of breaking strength/deformation, the peeling force between the ILM and retina, and identification of design parameters should be determined in future studies.

Our study showed that it is possible to laminate an artificial ILM with a PVA hydrogel thin film on an artificial retina, and we succeeded in constructing a fundus and then integrating the whole eye model for vitreoretinal surgery. This eye model could use three trocars for inserting an infusion needle, a light guide, and ILM microforceps, as used in actual surgery, and could be filled with water. When air bubbles entered the corneal part after filling with water, they could be removed with a needle. After completely filling the model with water, we could observe the fundus by applying a viscoelastic substance and inserting a contact lens. We therefore succeeded in developing a surgical simulator that could perform a sequence of operations related to the actual ILM peeling procedure. Because no special equipment other than a surgical simulator is required, ophthalmic microscopes and light sources can be used. For this reason, our simulator can be easily installed in existing medical offices and in dry/wet laboratories of manufacturers and dealers. We have shown that our eye surgery simulator has high validation for ILM peeling. However, it is necessary to introduce excellent skill evaluation and scoring methods such as those used in past studies using virtual reality [13–15]. Besides the video-based dynamic image analysis of the operator, installation of sensor functions in our simulator will allow us to propose a new design of surgical instruments and to construct a validation system.

Because this simulator has the modular structure of an artificial eyeball, exercises can be resumed by merely replacing the artificial eyeball alone. For this reason, we believe that it is possible to easily simulate other ocular surgeries by developing and exchanging artificial eyeballs for cataract and glaucoma surgery. Many surgical tasks can be simulated with our platform, which can incorporate different modules. In future studies, sensory evaluation of the ILM peeling task by a skilled physician should be evaluated, and the engineering parameters corresponding to this sensory evaluation should be identified. This will allow for the engineering parameters to potentially be correlated with sensory expressions, and the physician’s evaluation can then be quantitatively expressed by engineering parameters. Additionally, this may lead to the elucidation of a universal identification method of engineering parameters corresponding to sensory expressions, which can be used to develop future bionic models.

## Conclusions

We developed a novel ocular surgery simulator for ILM peeling using a realistic eye model installed with an artificial ILM. Because the natural ILM is a very weak, soft, thin film adhered to the retina, we succeeded in constructing a new-generation artificial ILM using a chemically cross-linked PVA hydrogel. We also installed the eye model onto an eye surgery simulator. As a result, we successfully developed a next-generation simulator for peeling the ILM underwater in a similar manner as in actual surgery. In the future, we anticipate improving our simulator with reference to the evaluations made by many vitreous surgeons.

## Supporting information

S1 Movie Example of peeling motion of a fabricated ILM in pure water. (MP4)

S2 Movie Example of surgical simulation of ILM peeling with an artificial eyeball mounted on the novel eye surgery simulator. We succeeded in completely simulating the ILM peeling procedure as in actual surgery.
(MP4)
